# E2F transcription factor 1 is involved in the phenotypic modulation of esophageal squamous cell carcinoma cells via microRNA-375

**DOI:** 10.1080/21655979.2021.1996510

**Published:** 2021-12-07

**Authors:** Pengfei Li, Huina Lv, Yongkai Wu, Ke Xu, Min Xu, Yegang Ma

**Affiliations:** a Department of Thoracic Surgery, Cancer Hospital of China Medical University, Liaoning Cancer Hospital and Institute, Shenyang, Liaoning, China; b Department of Ultrasound, Shengjing Hospital of China Medical University, Shenyang, Liaoning, China

**Keywords:** Esophageal squamous cell carcinoma, E2F1, microRNA-375, SESN3, the PI3K/AKT pathway

## Abstract

E2F family of transcription factors modulates multiple cellular functions associated with cell cycle and apoptosis. Here, we focused on the relevance of E2F1 to esophageal squamous cell carcinoma (ESCC) and identification of E2F1-mediated network in this study. Query of Gene Expression Omnibus database revealed that E2F1 was the core gene that was upregulated in ESCC. E2F1 downregulation inhibited ESCC cell activity. microRNA (miR)-375 was confirmed to be a downstream target of E2F1. E2F1 bound to miR-375 promoter and inhibited miR-375 transcription. Moreover, miR-375 inhibitor mitigated the repressive impacts of si-E2F1 on ESCC cells in part. Further study showed that sestrin 3 (SESN3) could interact with miR-375, and its knockdown annulled the stimulative effect of miR-375 inhibitor on ESCC development. Finally, E2F1 and SESN3 downregulation inhibited the phosphatidylinositol 3 kinase (PI3K)/AKT pathway activity in cells, while miR-375 inhibitor promoted PI3K/AKT pathway activation. These findings suggest that E2F1 inhibited miR-375 expression and promoted SESN3 expression to activate the PI3K/AKT pathway in ESCC.

## Introduction

Esophageal cancer remains a commonly diagnosed and fatal cancer in China in 2015 [1,2]. The 5-year overall survival rate of esophageal cancer is less than 20% in the world range [3], despite advance in surgical techniques, reduced perioperative mortality, and the development of multimodal therapies [4]. Esophageal squamous cell carcinoma (ESCC) represents a main histological kind of esophageal cancer worldwide, and tobacco and alcohol consumption, mutations of enzymes, achalasia, and caustic injury constitute the risk factors of ESCC [5]. In China, the survival rate is lower than 10% when diagnoses were made at advanced stages which can be as high as 85% once diagnosed earlier, and the mortality of ESCC can be decreased by early diagnosis and treatment [6]. Therefore, exploring new diagnostic biomarker and therapeutic options is essential for improvement in the overall survival of patients with ESCC.

E2F family of transcription factors modulates a vast network of cellular functions associated with cell cycle and apoptosis in cancers [7]. Overexpression of E2F1 has been detected in lower-grade glioma [8], glioblastoma, and lung, ovarian, breast, gastric, as well as colon cancers [9]. Moreover, a multiscale integrated analysis revealed that E2F1 showed excellent diagnostic and prognostic values, which may be a potential regulator in esophageal cancer [10]. Interestingly, E2F1 has been identified to bind to microRNA (miR)-1205 promoter and transcriptionally inhibit miR-1205 expression in laryngeal SCC [11]. Therefore, we wondered whether E2F1 serves as a transcription factor to repress the transcription levels of miRNAs in ESCC. miR-375 was commonly reduced and acted as a tumor suppressor that binds to various oncogenes in tumor cells, especially in human SCC [12]. For instance, miR-375 regulated metadherin (MTDH) to modulate cell proliferation in head and neck SCC [13]. Additionally, poor expression of miR-375 was linked to unsatisfactory outcome and metastasis in patients with head and neck SCC [14], indicating the clinical value of miR-375. More relevantly, miR-375 has been found to repress ESCC cell growth and invasion through binding to MTDH [15]. However, the upstream mechanism of miR-375 in ESCC has not been well-characterized to date. Our study aimed to investigate the expression as well as molecular mechanism of E2F1 in the development of ESCC. Furthermore, we hypothesized that E2F1 binds to miR-375 to regulate its downstream target expression, thereby regulating proliferation and apoptosis of ESCC cells. Our research might provide novel effective targets for the treatment and diagnosis of ESCC.

## Material and methods

### Data mining

Two data series consisting of 22 ESCC tissues and their matched normal tissue pairs were acquired from the GEO database (http://www.ncbi.nlm.nih.gov/geo). Among them, GSE20347 contains the tumor and normal adjacent tissues of 17 ESCC patients, and GSE17351 includes 5 pairs of primary ESCC and adjacent normal esophageal mucosa. Differentially expressed genes (DEG) between ESCC and normal tissues were screened out by GEO2R (http://www.ncbi.nlm.nih.gov/geo/geo2r/) with parameters set to *p*-value < 0.01 and log_2_fold change > 2, followed by heatmap plotting.

STRING (http://string-db.org/) database was utilized to determine related genes of E2F1. Data of the KEGG pathway enrichment analysis were obtained from KEGG (https://www.kegg.jp/kegg/rest/keggapi.html). TransmiR (http://www.cuilab.cn/transmir) was used to identify miRNAs interacting with E2F1. Pan-cancer analysis of E2F1 was conducted using Oncomine database. Cox analysis of E2F1 expression with ESCC patients’ characteristics was performed using the forestplot package in R (Version 3.6.3, NIH, Bethesda, MD, USA). The promoter sequence of miR-375 was obtained from UCSC website (https://genome.ucsc.edu/index.html), and the binding site between miR-375 and E2F1 was predicted by ALGEEN (http://alggen.lsi.upc.es/cgi-bin/promo_v3/promo). The target mRNAs of miR-375 and the binding site between miR-375 and SESN3 were acquired from the StarBase database (http://starbase.sysu.edu.cn/).

### Patients and tissue samples

From January 2015 to January 2017, we obtained 38 ESCC and adjacent tissues (3–5 cm away from the tumor tissues) from Cancer Hospital of China Medical University, Liaoning Cancer Hospital and Institute. The diagnosis was performed by three specialized pathologists. None of the patients received chemo-, immune- or radio-therapies before sample collection. After collection, the samples were instantaneously placed in liquid nitrogen and transferred to a − 80°C refrigerator until further analysis. Signed informed consent forms were acquired from patients. This item was endorsed by the ethics committee of Cancer Hospital of China Medical University, Liaoning Cancer Hospital and Institute.

### Immunohistochemistry

After conventional paraffin embedding, the human and mouse tumor tissues were cut into 4-μm histological sections, dewaxed with xylene, and hydrated with gradient density alcohol [16]. Antigen retrieval was conducted by boiling the sections for 10 min at 100°C in 10 mmol/L citric acid buffer (pH = 6.0). The sections were incubated with antibodies to E2F1 (1:500, sc-251, Santa Cruz Biotechnology Inc., Santa Cruz, CA, USA), SESN3 (1:100, H00143686-M02, Abnova, Walnut, CA, USA), and ki-67 (1:1000, sc-23,900, Santa Cruz) at 4°C overnight. Next, the sections were re-probed with HRP-coupled anti-mouse secondary antibody (1:2000, ab205719, Abcam Inc., Cambridge, UK) for 60 min at room temperature. Peroxidase activity was observed with diaminobenzidine tetrahydroxyl chloride solution (Vector Laboratories, Inc., Burlingame, CA, USA), and the sections were counter-stained with hematoxylin. After gradient density alcohol dehydration, xylene dewaxing, neutral resin (Merck Millipore, Darmstadt, Germany) sealing, the staining results were observed under a microscope (Nikon Instruments Inc., Melville, NY, USA).

### In situ hybridization

The miR-375 distribution in tissues was examined using an *in-situ* hybridization assay kit (Beyotime, Shanghai, China). The tissues were fixed in 4% paraformaldehyde for 0.5 h, and cultured with 0.25% acetic anhydride for 10 min before hybridization, followed by permeabilization with Triton X-100 for 10 min and proteinase K treatment to expose target nucleic acids. The sections were incubated with probe-free hybridization buffer for 120 min at ambient temperature to block nonspecific binding. Hybridization was conducted using a synthetic miR-375 probe. The sections were treated with 10 μL hybridization solution overnight at 42°C, washed with gradient concentrations of saline citrate (20 min each) at 37°C, and sealed with 3% BSA for 0.5 h at 37°C. Next, the sections were cultured with anti-digoxigenin anti-serum alkaline phosphatase complex overnight at 4°C. Finally, diaminobenzidine color development solution was added, and the section was placed in Tris-ethylenediamine tetraacetic acid solution for 10–30 min to terminate the reaction. After gradient density alcohol dehydration, xylene dewaxing, neutral resin (Merck Millipore) sealing, and the staining results were observed under a microscope (Nikon).

### RT-qPCR analysis

Total RNA was isolated from the cells with the help of TRIzol (Thermo Fisher Scientific Inc., Waltham, MA, USA) as per the protocol. cDNA was produced with oligonucleotide primers in a 20 μL reaction system using iScript (Bio-Rad Laboratories, Hercules, CA, USA). Real-time PCR of cell line samples was conducted using the Universal Probe Library monochrome probe from the Roche LightCycler 480 system (Roche Diagnostics, Basel, Switzerland) on 5 μL diluted cDNA. The change in expression was measured with the help of the 2^−ΔΔCq^ method [17]. The mentioned primer sequences are exhibited in Table 1.

**Table 1.** PCR primer sequences for selected gene

Gene ID	Sequence (5’-3’)
E2F1	Forward: TCTCCGAGGACACTGACA
	Reverse: GTTCTCCGAAGAGTCCAC
miR-375	Forward: GGCTCTAGAGGGGACGAAGC
	Reverse: GGCAAGCTTTTTCCACACCTCAGCCTTG
SESN3	Forward: CCGCCAGTAACTATCATACATGCG
	Reverse: GAGGATGTTGACACAACCATGCTG
U6	Forward: GCTTCGGCAGCACATATACTAAAAT
	Reverse: CGCTTCACGAATTTGCGTGTCAT
GAPDH	Forward: GGAGCGAGATCCCTCCAAAAT
	Reverse: GGCTGTTGTCATACTTCTCATGG

**Note**: miR, microRNA; SESN3, sestrin 3; GAPDH, glyceraldehyde-3-phosphate dehydrogenase.

### Culture and treatment of cells

An ESCC cell line KY150 and human esophageal epithelial cells HET1A were from EK Bioscience Biotechnology (Shanghai, China). EC109, EC9706, and YES2 from BeNa Culture Collection (Beijing, China) were plated in RPMI-1640 plus 10% FBS at 37°C and 5% CO_2_ (Thermo Fisher Scientific). The medium was refreshed at an interval of 24 h. When the cell confluence reached 80%, the cells were detached with 0.25% of trypsin. The miR-375 inhibitor (miR-375 inh) and its corresponding miR-375 control (miR-375 con), siRNA against E2F1 and SESN3 were from RiboBio (Guangzhou, Guangdong, China). ESCC cells were plated overnight and transiently transfected using Lipofectamine 2000 (Thermo Fisher scientific). The cells were cultivated for 24 or 48 h prior to following experiments.

### CCK-8 analysis

The cells were resuspended in PBS and plated in a 96-well plate at 2 × 10^3^ cells/well [18]. Following a one-night culture at 37°C with 5% CO_2_, CCK-8 (10 μL, Beyotime) was supplemented to each well and cultured at 37°C. After 3 d, the OD value was read at 450 nm under a microplate reader (Bio-Rad, Hercules, CA, USA). Median inhibition concentration (IC50) values were analyzed from the dose response curves of GraphPad Prism 8.0 software.

### Transwell assays

Cell motility was studied by cell invasion and migration [19]. For the migration assay, EC109 and YES2 cells (1 × 10^4^ cells/well) were suspended in serum-free DMEM and supplemented to the apical chamber. For the invasion assay, 1 × 10^4^ cells were plated into Matrigel-coated chambers. DMEM plus 10% FBS (500 μL) was plated to the basolateral chamber, and allowed to culture for 24 h. The cells in the basolateral chamber were stained with 0.1% crystal violet. The number of migratory or invasive cells was measured under a 200x magnification microscope (Carl Zeiss, Oberkochen, Germany) in three random fields of view.

### Flow cytometric analysis

Annexin V-FITC Apoptosis Detection kits (Abcam) were utilized for apoptosis examination. The cell samples were stained with 5 μL Annexin V-FITC and 5 μL PI for 0.5 h at ambient temperature in darkness and loaded onto the BD FACSCalibur Flow Cytometry System (BD Biosciences, San Jose, CA, USA).

### Chromatin immunoprecipitation (ChIP)

Briefly, the 1 × 10^7^ ESCC cells were cross-linked with 1% formaldehyde and resuspended in lysis buffer [20]. The cell lysates were sonicated on ice, and the average length for DNA fragment was 500 bp. Following centrifugation, IP was conducted overnight in ChIP dilution buffer with antibodies specific to IgG or E2F1 (1:800, sc-251, Santa Cruz Biotechnology). The samples were incubated with protein A agarose/salmon sperm DNA (Merck Millipore) slurry at 4°C for 2–4 h with agitation. The antibody-agarose complexes were centrifuged, and the immunoprecipitated fractions were eluted. The DNA was de-crosslinked by a 4-h incubation at 65°C, recovered, and precipitated by phenol/chloroform extraction. The enrichment of specific sequences was assessed by qPCR.

### Luciferase assay

The SESN3 3ʹuntranslated region (3ʹUTR) sequence containing the miR-375 binding site was subcloned into pmirGLO luciferase reporter vectors (Promega Corporation, Madison, WI, USA) to produce a wild-type (WT) vector, while the corresponding mutant (MT) was produced by mutating the binding site. The luciferase reporter vector was co-transfected into EC109 cells with miR-375 con or miR-375 inh, and the luciferase reporter system (Promega) was utilized for luciferase activity analysis 24 h after transfection.

### Nude mice tumorigenesis assay

Our research was permitted by the Ethical Committee of Cancer Hospital of China Medical University, Liaoning Cancer Hospital and Institute. Animal studies were executed in compliance with the ARRIVE guidelines. BALB/c nude mice (4 weeks old, male, n = 30) from the Shanghai Lab Animal Research Center (Shanghai, China) were acclimatized for 7 d and randomly allocated into six groups (n = 5). EC109 cells (2 × 10^6^ cells) stably transfected with low expression plasmids were used for subcutaneous injection in nude mice. The length and width of tumors in nude mice were assessed every week to determine tumor volume as volume (mm^3^) = width^2^ × length/2. Euthanasia was performed using intraperitoneal injection of 1% pentobarbital sodium at 4 weeks after injection, and tumors were harvested for weighing and molecular analysis.

### Western blot analysis

RIPA buffer (pH = 8.0) including 150 mM NaCl, 0.1% SDS, 50 mM Tris-HCl and 1% NP-40 was added to the proteinase inhibitor (Roche Diagnostics, Co., Ltd., Rotkreuz, Switzerland) and used to obtain whole cell lysates. The proteins were examined using a BCA kit (Thermo Fisher) and separated by SDS-PAGE. The proteins were transferred onto PVDF membranes (0.45 µm, Millipore Corp, Billerica, MA, USA), sealed with 5% BSA for 60 min at 25°C, and hybridized at 4°C with primary antibodies to MMP2 (1:1000, MA5-14,186, Invitrogen Inc., Carlsbad, CA, USA), MMP9 (1:2000, ab58803, Abcam), Bax (1:1200, sc-7480, Santa Cruz Biotechnology), PI3K (1:1,800, sc-8010, Santa Cruz Biotechnology), p-AKT (1:1,500, ab81283, Abcam), AKT (1:1,300, sc-5298, Santa Cruz Biotechnology), and GAPDH (1:3,000, G8795, Sigma-Aldrich, St Louis, MO, USA) for one night. The blots were incubated with secondary antibodies for 60 min at 25°C. Finally, the protein bands were detected using an ECL kit (Thermo Fisher Scientific). Densitometric analysis was performed using IPP 6.0 (Image-Pro Plus 6.0).

## Statistics

All analyses were implemented using SPSS (22.0, IBM Corp. Armonk, N.Y., USA). The data were recorded as means ± SD. Paired *t*-test was carried out for two-group comparison, and ANOVA followed by Tukey’s post hoc test was utilized to compare differences when the number of groups was greater than two. Correlations between genes were calculated using Person’s correlation analysis. All differences were considered statistically significant when *p* < 0.05.

## Results

In our results, we found that expressions of E2F1 was upregulated in ESCC, and silencing of E2F1 reduced the expression of MMP-associated proteins, cell proliferation, invasion and migration in ESCC cells. We also found that miR-375 was the downstream target of E2F1, and SESN3 was the putative target of miR-375 in ESCC. E2F1 promoted the SESN3 expression and the PI3K/AKT pathway through decreasing the transcription of miR-375.

### mRNA profiling in human ESCC

**Figure 1.**
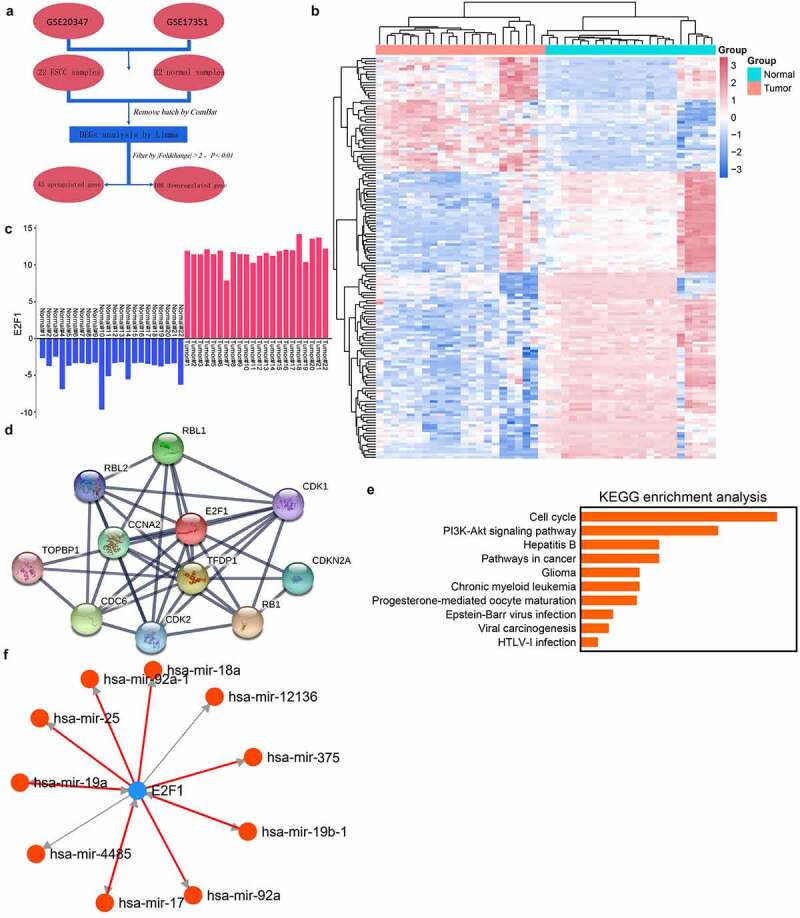
E2F1 is upregulated in human ESCC. (a) DEG in ESCC through GEO database. (b) Demonstration of DEG by a heatmap. (c) E2F1 expression in GSE20347 and GSE17351 databases, where Tumor/Normal 18–22 were from GSE17351. (d) E2F1-related genes in the PPI network. (e) KEGG pathway enrichment analysis of the E2F1 gene cluster. (f) Analysis of E2F1-regulated downstream miRNAs

To identify candidate genes in ESCC, we implemented bioinformatics analyses using published datasets (Figure 1a). Briefly, two data arrays consisting of 22 ESCC tissues and adjacent tissues were acquired from the GEO database and normalized. This genome identified 43 significantly upregulated genes and 108 significantly downregulated genes in ESCC tissues (Figure 1b). E2F1 was the most upregulated molecule in both databases, signifying that it may play a part in the ESCC progression (Figure 1c). To study the biological function of E2F1, genes with expression closely related to this molecule (absolute Pearson correlation coefficient > 0.9) were chosen as input for the KEGG pathway analysis (Figure 1d). These genes were mostly enriched in the PI3K/AKT pathway (Figure 1e). To further investigate the underlying mechanism of E2F1, TransmiR was used to identify molecules interacting with E2F1. The miRNAs with the top ten binding-site scores were selected as candidate downstream genes (Figure 1f).

### Upregulation of E2F1 in ESCC is related to poor prognosis

**Figure 2.**
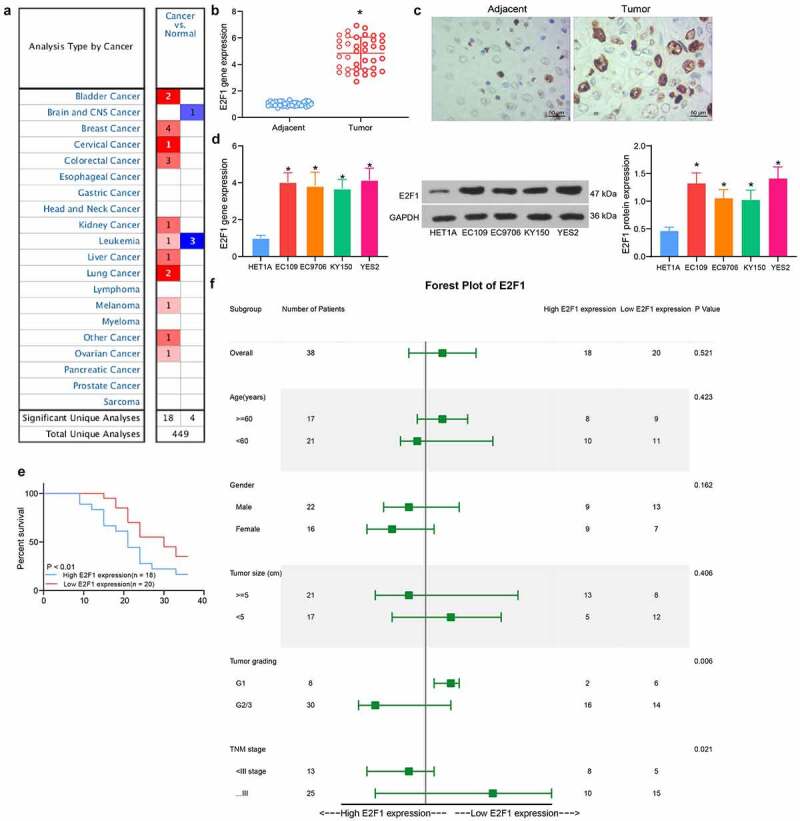
Upregulation of E2F1 in ESCC is correlated with tumor grading and poor prognosis. (a) Detection of E2F1 expression in pan-cancer by Oncomine database (The complete picture is shown in Fig. S1). (b) Detection of E2F1 expression between ESCC and normal tissues by RT-qPCR. (c) Immunohistochemical detection of the distribution of E2F1 in tissues. (d) E2F1 mRNA and protein expression in ESCC cells by RT-qPCR and western blot. (e) The prognosis of E2F1 in ESCC patients was analyzed using Kaplan-Meier analysis. (f) Cox analysis of the correlation between E2F1 and pathological characteristics of ESCC patients. The data were recorded as means ± SD. Paired *t*-test was utilized for two-group comparison (panel B), and one-way ANOVA followed by Tukey’s post hoc test was applied to compare differences when the number of groups was greater than two (panel D). *p < 0.05

Pan-cancer analysis of E2F1 revealed that E2F1 was commonly overexpressed in cancerous tissues (Figure 2a). We assessed the E2F1 expression in 38 pairs of ESCC tissues collected by RT-qPCR, and the data demonstrated that the E2F1 expression was increased in ESCC tissues (Figure 2b). Immunohistochemical staining for E2F1 also showed strong positivity of E2F1 in ESCC tissues (Figure 2c). After that, we assessed the E2F1 mRNA and protein expression in EC109, EC9706, KY150 and YES2 cells. It was showed that E2F1 expression was promoted in ESCC cells at both mRNA and protein levels versus normal ESCC cells HET1A (Figure 2d). According to the mean value of E2F1 expression, we distinguished patients with high E2F1 expression from those with low E2F1 expression, and observed that the ESCC patients with high E2F1 expression suffered from shorter survival time (Figure 2e). In addition, E2F1 overexpression was tightly linked to tumor grade and TNM stages (Figure 2f). All these findings propose that E2F1 is involved in the ESCC progression.

### Depletion of E2F1 inhibits ESCC cell activity

**Figure 3.**
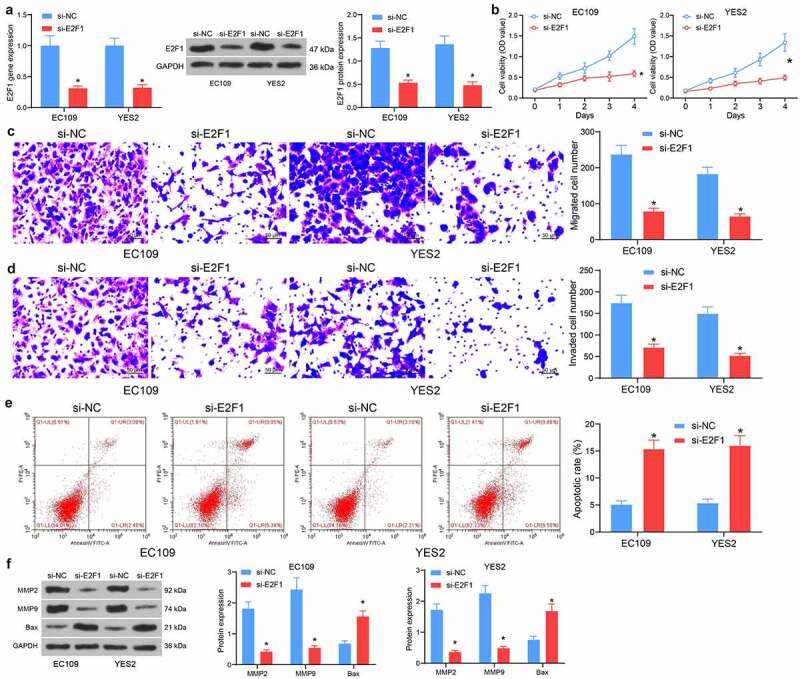
Depletion of E2F1 hampers proliferation and invasiveness of ESCC cells. (a) The E2F1 mRNA and protein expression in cells in response to si-NC or si-E2F1 by RT-qPCR and western blot. (b) The proliferative activity of ESCC cells by CCK8 assay. (c) The migratory activity of ESCC cells by Transwell assay. (d) The invasive activity of ESCC cells by Transwell assay. (e) The apoptotic activity of ESCC cells by flow cytometry. (f) The protein expression of MMP2, MMP9 and Bax in cells in response to si-NC or si-E2F1 by western blot. The data were recorded as means ± SD. Two-way ANOVA followed by Tukey’s post hoc test was applied to compare differences when the number of groups was greater than two. *p < 0.05

To study the specific function of E2F1 in ESCC progression, EC109 and YES2 cells with the relative high expression of E2F1 were delivered with si-NC or si-E2F1. After transfection, the mRNA and protein expression of E2F1 was remarkably reduced (Figure 3a). In terms of proliferation, silencing of E2F1 appreciably constrained cell proliferation (Figure 3b). Similarly, knockdown of E2F1 reduced the number of migrated EC109 and YES2 cells (Figure 3c). Furthermore, knockdown of E2F1 resulted in a decreasing trend of cell invasion activity (Figure 3d). In contrast, E2F1 knockdown increased the proportion of apoptotic cells and elevated apoptotic activity (Figure 3e). Detection of the expression of MMP- and apoptosis-associated proteins in cells showed decreased protein levels of MMP2 and MMP9 as well as elevated Bax after E2F1 downregulation (Figure 3f). Our results suggest that silencing of E2F1 inhibits the biological activity of ESCC cells.

### E2F1 potentiates ESCC progression through binding to miR-375

**Figure 4.**
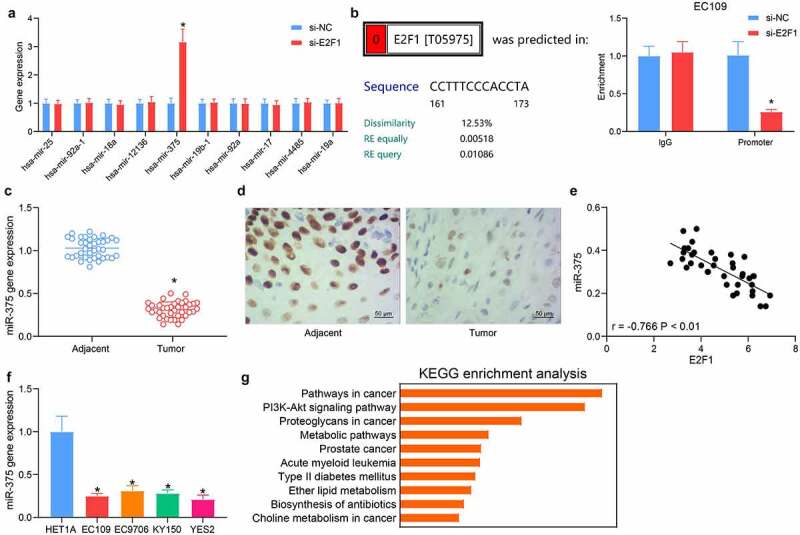
Direct regulation of miR-375 by E2F1 in ESCC cells. (a) Detection of possible downstream miRNA expression in cells in response to si-NC or si-E2F1 by RT-qPCR. (b) The relationship between E2F1 and miR-375 by ChIP experiments. (c) The miR-375 expression in ESCC tissues by RT-qPCR. (d) The miR-375 expression in ESCC tissues by in situ hybridization. (e) The relationship between E2F1 and miR-375 analyzed using Person’s correlation analysis. (f) The miR-375 expression in ESCC cells. (g) KEGG pathway enrichment analysis was performed on miR-375 target genes. The data were recorded as means ± SD. Paired *t*-test was applied for two-group comparison (panel C), and one-way (panel F) or two-way ANOVA (panels A & B) followed by Tukey’s post hoc test was utilized to compare differences when the number of groups was greater than two. *p < 0.05

Based on the results in Figure 1f, we detected the expression patterns of downstream miRNAs in EC109 cells poorly expressing E2F1. miR-375 was significantly elevated with the knockdown of E2F1, indicating that miR-375 might be a possible downstream target of E2F1 in ESCC cells (Figure 4a). After predicting the binding sites between miR-375 and E2F1, the interaction between E2F1 and miR-375 was verified by ChIP assay. Downregulation of E2F1 in EC109 cells resulted in a significant reduction of E2F1 protein bound to the miR-375 promoter relative to control IgG, indicating the possible binding relation between E2F1 and miR-375 (Figure 4b). Measurement of miR-375 expression in tissues of ESCC patients using RT-qPCR demonstrated that miR-375 was reduced in ESCC tissues (Figure 4c). In situ hybridization showed weak staining of miR-375 in patients’ tumor tissues (Figure 4d). To detect the correlation between E2F1 and miR-375 in patients, miR-375 and E2F1 expression in ESCC tissues of 38 patients were listed and subjected to Person’s correlation analysis, with confidence intervals chosen as 95% and R-values as curve fit, showing a significant negative correlation between miR-375 and E2F1 (Figure 4e). In addition, reduced miR-375 expression was noted in all ESCC cells analyzed (Figure 4f). The enrichment analysis of miR-375 target genes revealed the same results as those of E2F1. miR-375 target genes were principally enriched in the PI3K/AKT pathway (Figure 4g), showing that miR-375 is a downstream target of E2F1.

### SESN3 is a putative target of miR-375

**Figure 5.**
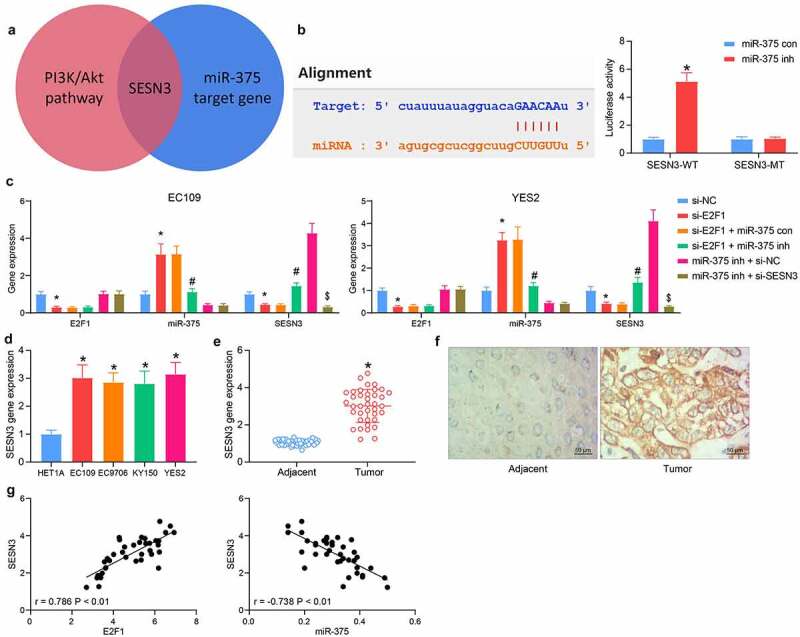
SESN3 is a candidate target of miR-375 in ESCC. (a) The screening for miR-375 downstream genes using a Venn diagram. (b) the interrelationship between miR-375 and SESN3 by luciferase assay. (c) Detection of E2F1, miR-375, and SESN3 expression in cells in response to si-E2F1 + miR-375 inh or miR-375 inh + si-SESN3 by RT-qPCR. (d) The SESN3 mRNA expression in ESCC cells by RT-qPCR. (e) The SESN3 mRNA expression in ESCC tissues by RT-qPCR. (f) Distribution of SESN3 in tissues by immunohistochemical staining. (g) Person’s correlation analysis of the correlation between E2F1 and SESN3 or miR-375 and SESN3. The data were recorded as means ± SD. Paired *t*-test was applied for two-group comparison (panel E), and one-way (panel D) or two-way ANOVA (panels B & C) followed by Tukey’s post hoc test was applied to compare differences when the number of groups was greater than two. *#$*p* < 0.05

The miR-375 target genes with the most enrichment pathways and biological processes were overlapped with the genes enriched in the PI3K/AKT pathway, and SESN3 was observed to be the only result (Figure 5a). Luciferase assay was then carried out to verify the binding between miR-375 and SESN3 mRNA. miR-375 inhibitor significantly increased the fluorescence intensity of SESN3-WT at the predicted binding site (Figure 5b). The SESN3 expression in cells poorly expressing E2F1 was significantly reduced, which was elevated by miR-375 inhibitor. The expression of both E2F1 and miR-375 was not changed by the co-transfection of miR-375 inhibitor and si-SESN3 relative to miR-375 inhibitor + si-NC (Figure 5c). Examination of SESN3 expression in ESCC tissues and cells revealed that SESN3 was significantly elevated in the ESCC cells (Figure 5d) and tissues (Figure 5e). Immunohistochemical staining showed consistent results in ESCC tissues (Figure 5f). SESN3 showed a significant positive correlation with E2F1 expression and a converse correlation with miR-375 expression in ESCC tissues (Figure 5g), indicating SESN3 was a candidate target of miR-375 in ESCC.

### E2F1/miR-375/SESN3 axis regulates the malignant invasiveness of ESCC cells

**Figure 6.**
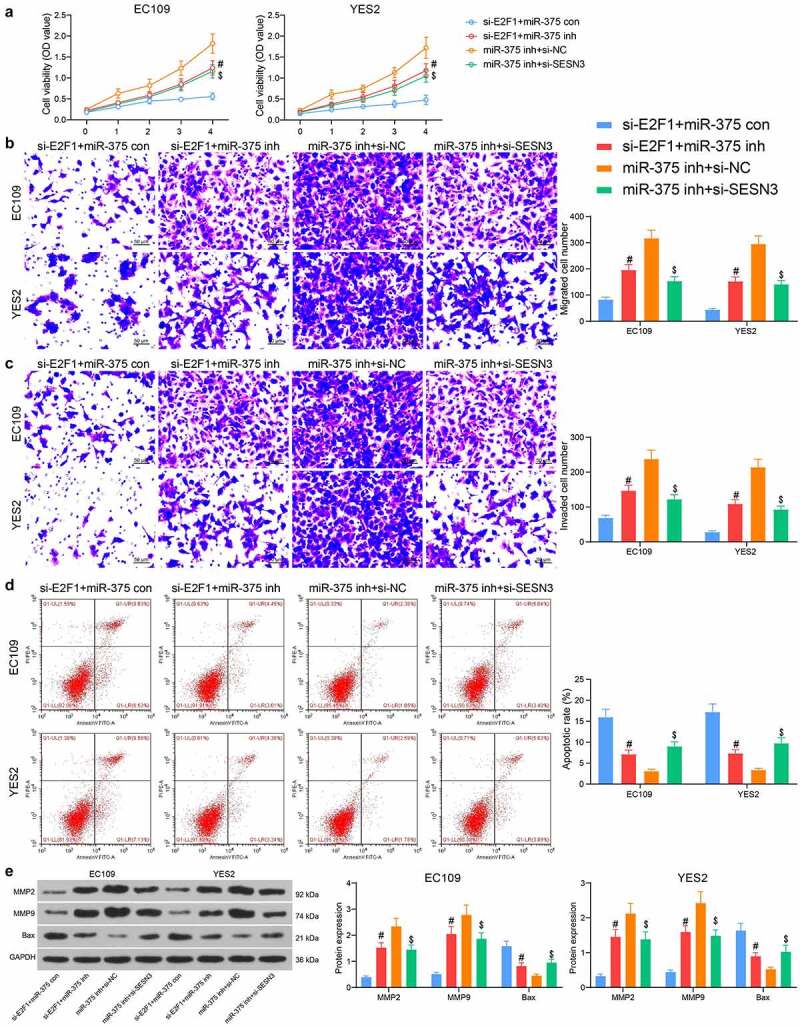
E2F1 regulates ESCC cell malignant aggressiveness through the miR-375/SESN3 axis. (a) The proliferative activity of ESCC cells by CCK8 assay. (b) The migratory activity of ESCC cells by Transwell assay. (c) The invasive activity of ESCC cells by Transwell assay. (d) The apoptotic activity of ESCC cells by flow cytometric analysis. (e) The protein expression of MMP2, MMP9 and Bax in cells by western blot. The data were recorded as means ± SD. Two-way ANOVA followed by Tukey’s post hoc test was applied to compare differences when the number of groups was greater than two. #$*p* < 0.05

EC109 and YES2 cells were co-transfected with si-E2F1 + miR-375 inh, miR-375 inh + si-SESN3 or their respective controls (si-E2F1 + miR-375 con and miR-375 inh + si-NC). The downregulation of miR-375 contributed to a significant rise in cell proliferation in the presence of si-E2F1, while the downregulation of SESN3 similarly inhibited the effect of miR-375 inhibitor, resulting in a significant decline in cell proliferation activity (Figure 6a). Migrated cell counts showed that lowering miR-375 enhanced the migratory function of ESCC cells, while downregulation of SESN3 diminished the migration of ESCC cells (Figure 6b). Similarly, transfection of miR-375 inhibitor increased the cell invasion, and conversely si-SESN3 inhibited the effect of miR-375 inhibitor to decrease the cell invasion (Figure 6c). The detection of apoptotic activity revealed that miR-375 inhibitor reduced the number of apoptotic cells in ESCC cells, while SESN3 downregulation increased the number of apoptotic cells (Figure 6d). After that, western blot showed that miR-375 inhibitor elevated expression of MMP2 and MMP9, but reduced expression of the pro-apoptotic protein Bax. In contrast, si-SESN3 inhibited MMP2 and MMP9, and elevated Bax expression in the presence of miR-375 inhibitor (Figure 6e).

### E2F1/miR-375/SESN3 axis regulates the tumor growth of ESCC cells in vivo

**Figure 7.**
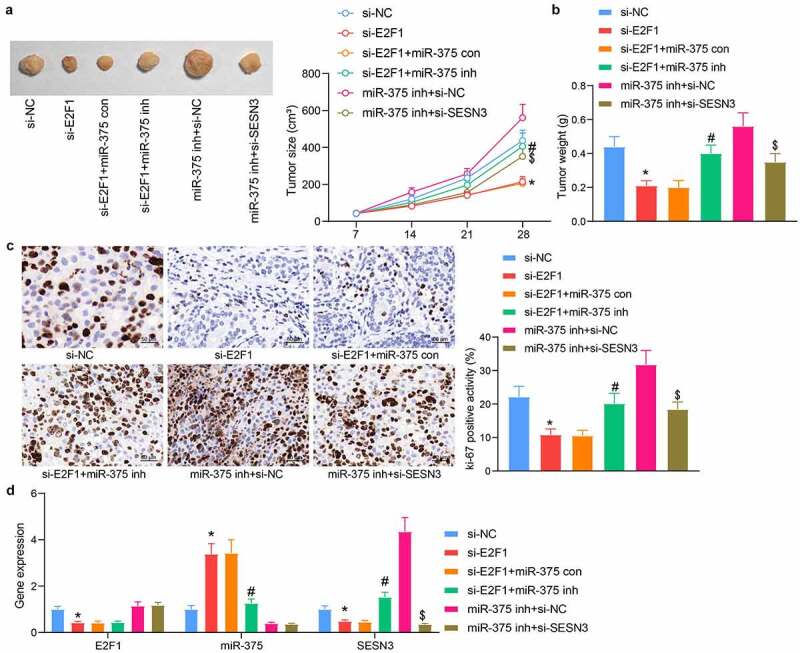
E2F1 regulates tumor growth in ESCC through the miR-375/SESN3 axis. (a) The tumorigenic activity of ESCC cells by *in vivo* tumorigenic assay. (b) Tumor weight detection of tumor formation in mice. (c) Immunohistochemical detection of ki-67 expression changes in tumor tissue. (d) Detection of E2F1, miR-375, and SESN3 in tumor tissues by RT-qPCR. The data were recorded as means ± SD (n = 5). Two-way ANOVA followed by Tukey’s post hoc test was applied to compare differences when the number of groups was greater than two. *#$*p* < 0.05

Highly active EC109 cells were injected into mice to verify the interrelationship between E2F1, miR-375, and SESN3. It was found that downregulation of E2F1 significantly hampered the volume of xenograft tumors, whereas miR-375 inhibitor enlarged the volume of xenograft tumors in mice. Similarly, si-SESN3 significantly reduced the tumorigenic activity of cells compared with the downregulation of miR-375 alone (Figure 7a). Our examination of tumor weight also illustrated these trends (Figure 7b). Immunohistochemical staining results showed that E2F1 knockdown prominently decreased the numbers of ki-67^+^ proliferating cells, which was mitigated by miR-375 inhibitor. In contrast, SESN3 downregulation reversed the effect of miR-375 downregulation and decreased tumor proliferation (Figure 7c). We also examined the expression profile of E2F1, miR-375, and SESN3 in tumor tissues derived from nude mice and found consistent results as Figure 5c in cells (Figure 7d). Therefore, we concluded that the E2F1/miR-375/SESN3 gene axis also functioned *in vivo*.

### PI3K/Akt pathway is mediated by the E2F1/miR-375/SESN3 axis

**Figure 8.**
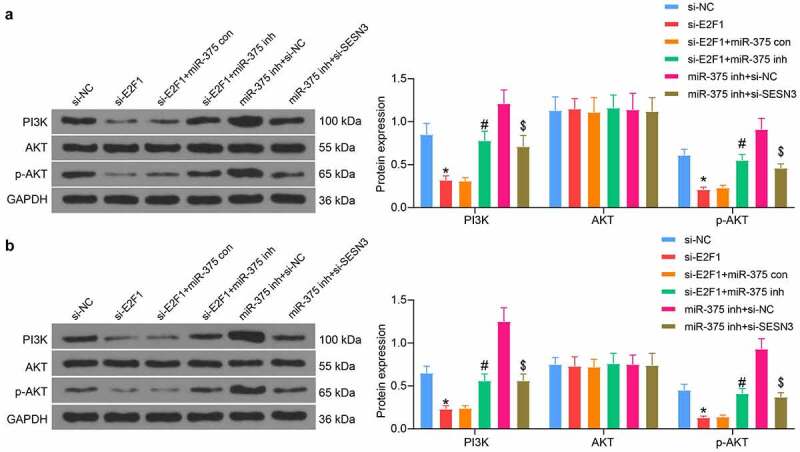
E2F1/miR-375/SESN3 axis mediates the PI3K/AKT signaling activity in ESCC cells. (a) PI3K/AKT pathway activation in ESCC cells by western blot. (b) PI3K/AKT pathway activation in tumor tissues from mice by western blot. The data were recorded as means ± SD. Two-way ANOVA followed by Tukey’s post hoc test was applied to compare differences when the number of groups was greater than two. *#$*p* < 0.05

Since the PI3K/AKT pathway was shown to be an enriched pathway in bioinformatics prediction, we examined the changes in the PI3K/AKT pathway activities in ESCC cells and tissues. E2F1 downregulation was found to decrease the extent of AKT phosphorylation and PI3K expression. miR-375 inhibitor restored the PI3K/AKT pathway activity, while SESN3 downregulation constrained the effect of miR-375 inhibitor and blocked the PI3K/AKT pathway activation again (Figure 8a). We also found this trend in xenograft tumors in mice (Figure 8b). Thus, we identified the PI3K/AKT pathway as a downstream pathway mediated by the E2F1/miR-375/SESN3 axis in ESCC.

## Discussion

E2F1 is a vital player in the events of DNA replication and cell cycle progression through its G1/S transcriptional activity [21]. In this study, we reported that E2F1 was not only overexpressed in patients with ESCC but also closely correlated with clinicopathological parameters. We further demonstrated that E2F1 silencing hampered ESCC cell growth and invasiveness *in vitro* and *in vivo* using loss-of-function assays. Moreover, increased E2F1 expression reduced the expression of miR-375 to induce SESN3, thereby activating the PI3K/AKT pathway in ESCC cells. These results implied that E2F1 might be an oncogene in ESCC and function as a possible therapeutic target.

Firstly, using integrated GEO database query and PPI network establishment, we successfully identified the core gene E2F1 and its associated PI3K/AKT signaling and ten miRNAs in ESCC. Moreover, E2F1 overexpression was found in ESCC tissues and cells relative to their counterparts, which predicted dismal prognosis of patients. Consistently, overexpression of E2F1 observed in both osteosarcoma and colorectal cancer tissues were related to TNM stage and distant metastasis [22,23]. Lee *et al*. reported that the overexpression of E2F1 enhanced bladder cancer cell colony formation, migration, and invasiveness, and depletion of E2F1 blocked cell invasion and reduced tumor size *in vivo* [24]. These findings were largely in line with our observations in ESCC cells.

Among the ten miRNAs screened out to be the downstream target of E2F1, only miR-375 was restored following E2F1 knockdown, which was chosen as our target. Likewise, in renal carcinoma, the expression of miR-520c-3p, miR-372-3p and miR-373-3p was evidently upregulated when E2F1 was downregulated, signifying that E2F1 might be a potential upstream modulator of these three miRNAs [25]. Our further ChIP-qPCR assay validated the direct interaction between miR-375 and E2F1. Previously, miR-375 has been indicated to be regulated by circular RNA and long noncoding RNA (lncRNA) in ESCC and tongue squamous cell carcinoma, respectively [26,27]. However, there is little information concerning the interactions between miR-375 and transcription factors, which highlights the novelty of our research. Isozaki *et al*. showed that the miR-375 expression was drastically reduced in clinical ESCC samples versus adjacent normal tissues [28]. Moreover, enforced expression of miR-375 hindered cell proliferation, colony formation, migration as well as invasion in ESCC and laryngeal squamous cell carcinoma [29–31]. In addition, miR-375 upregulation is sufficient to slow down tumor cell growth and metastasis *in vivo* in ESCC [32]. Also, a review has addressed that miR-375 stands for a hopeful direction for exploring targeted therapies because of its capability to hinder cancer cell growth [33].

SESNs are a group of highly conserved stress-responsive proteins and transcriptionally controlled by p53 and forkhead transcription factor (FOXO) [34]. It is believed that SESN3 represents a core mediator of ROS downstream of the AKT signaling pathway and FOXO transcriptional factors [35]. Also, it was identified as the downstream target of miR-675 in liver cancer [36]. The bioinformatics prediction conducted in the present study also verified that SESN3 was not only a gene enriched in the PI3K/AKT signaling, but also a putative target of miR-375 in ESCC cells. Interestingly, suppressed proliferation of ESCC cells by lncRNA SNHG22 knockdown was offset by SESN3 overexpression [37], indicating the oncogenic role of SESN3 in ESCC. In the present study, we observed the decline in SESN3 expression in ESCC cells upon E2F1 depletion, which was restored partially by miR-375 inhibitor. By contrast, mRNA levels of SESN3 were found unaffected by the loss of E2F1 in murine Embryonic fibroblasts [38]. We attributed this discrepancy to the presence of miR-375 in ESCC cells with E2F1 depletion. Our rescue experiments, consistently, showed that silencing of SESN3 mitigated the stimulative effects of miR-375 inhibitor on ESCC biological activities and tumor growth *in vivo*. The possible linkage between E2F1 and the PI3K/AKT signaling has been recently appreciated in head and neck squamous cell carcinoma [39]. Furthermore, overexpression of miR-375 led to the growth inhibition for colorectal cancer cells by governing the PI3K/AKT signaling [40]. The western blot results on both ESCC cells and tumor tissues revealed that silencing of E2F1 blocked the PI3K/AKT signaling pathway, which was restored by miR-375 inhibitor. While the SESN3 knockdown further contributed to the pathway deficit. However, this study has some limitations. For instance, only 38 pairs of tissues were obtained in this study. Therefore, we need to include more patients to make our conclusions more accurate and reliable. Further studies are also needed to focus on the involvement of the PI3K/AKT pathway in E2F1/miR-375/SESN3-mediated ESCC progression with PI3K/AKT inhibitors.

## Conclusion

**Figure 9.**
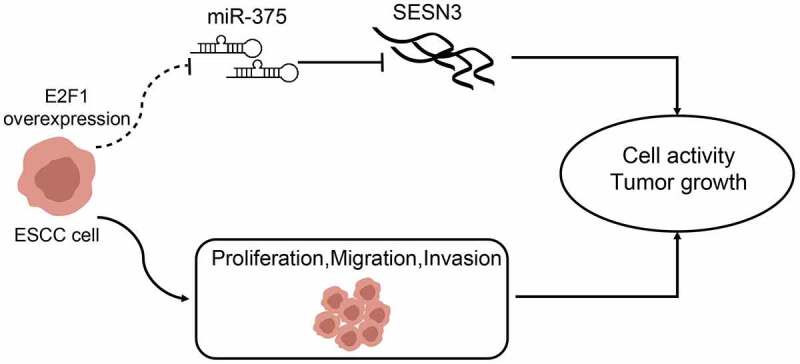
A graphical abstract for the study. Overexpression of E2F1 in ECSS cells elevated cell proliferation, migration, and invasion by elevating miR-375-controlled SESN3

Taken together, our results demonstrate a regulation mechanism between the transcription factor E2F1 and miR-375 in ESCC progression (Figure 9). Clinically, E2F1 displays a remarkable potential value for ESCC prognosis, which has improved our understanding of the molecular pathology of E2F1, thus providing a possible therapeutic target for ESCC treatment.

## Supplementary Material

Supplemental Material

## Data Availability

All the data generated or analyzed during this study are included in this published article.

## Highlights

1. E2F1 is highly expressed in ESCC.
2. E2F1 inhibits the expression of miR-375.
3. miR-375 binds to SESN3.
4. E2F1/miR-375/SESN3 axis is involved in ESCC progression.
5. E2F1/miR-375/SESN3 axis mediates the PI3K/AKT pathway.
