# Supplementary figures and images for: E2F transcription factor 1 is involved in the phenotypic modulation of esophageal squamous cell carcinoma cells via microRNA-375
Source: Bioengineered. 2021 Dec 7;12(2):10047–62. doi: 10.1080/21655979.2021.1996510 (PMC8809976; doi:10.1080/21655979.2021.1996510)

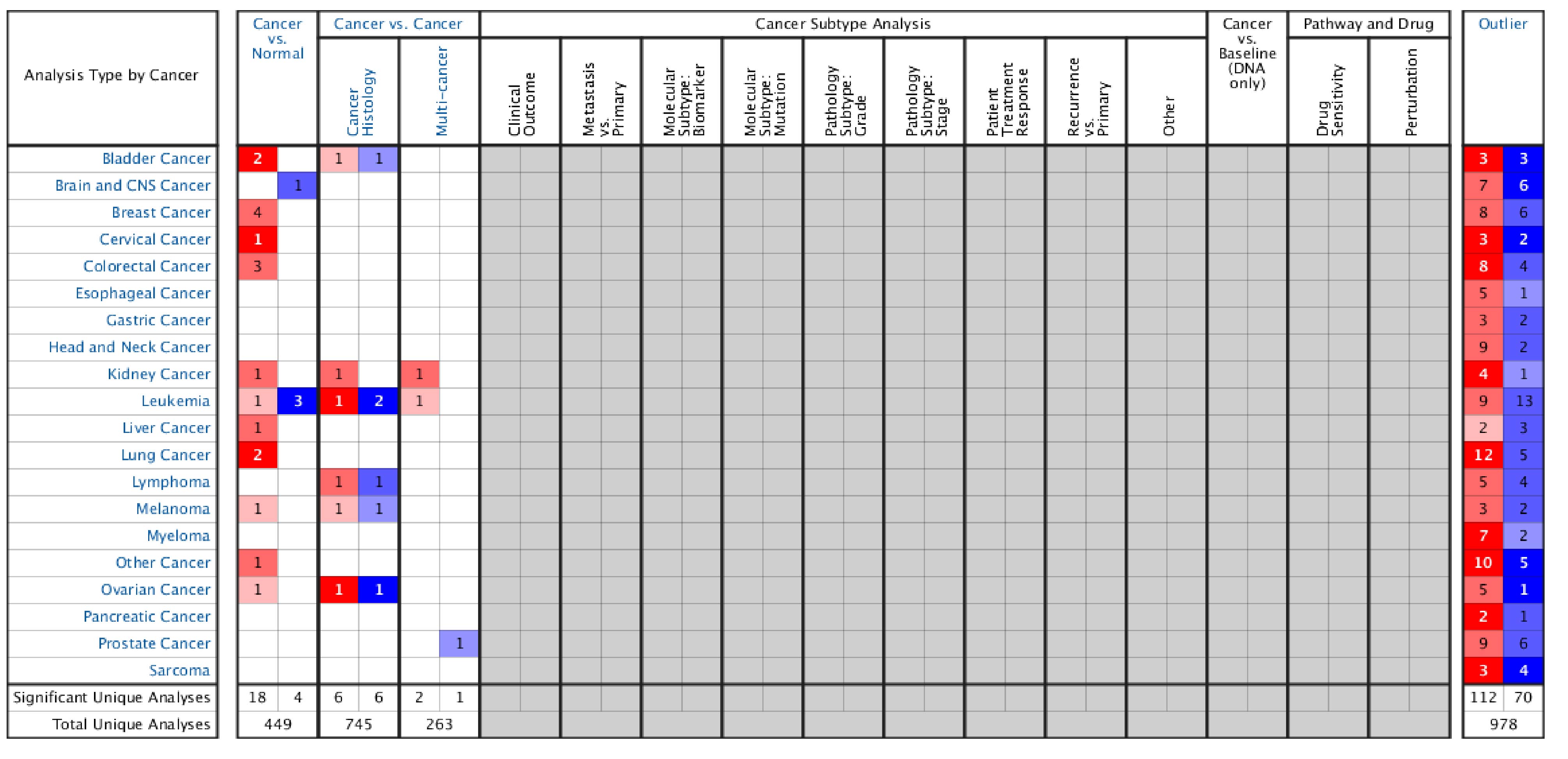

Supplement: Supplemental Material [file KBIE_A_1996510_SM6466.jpg]
